# In Situ ZIF-8-Coated Copper Laminate System for Fluid-Phase Adsorptive Separation

**DOI:** 10.1021/acsami.5c04227

**Published:** 2025-05-15

**Authors:** Ravi Sharma, Shiara Uyttersprot, Gino V. Baron, Joeri F. M. Denayer

**Affiliations:** Chemical Engineering Department, 70493 Vrije Universiteit Brussel, Brussels B-1050, Belgium

**Keywords:** ZIF-8, structured adsorbent, LEGO, laminate system, adsorptive separation, thermal swing adsorption

## Abstract

The use of structured adsorbents is emerging as a promising approach for adsorptive separation processes, and several ex situ structuring routes like extrusion, three-dimensional (3D) printing, and coating over substrates have been extensively investigated. However, in situ growth of adsorbents such as metal–organic frameworks (MOFs) on metal laminates remains underexplored. This study introduces a novel laminate system, where aluminum pieces, inspired by the “LEGO” concept, were designed through CNC milling and used to fabricate embossed/dented copper laminates. These laminates were then coated with ZIF-8 crystals (ZIF-8@Cu) via a direct in situ coating method at room temperature, resulting in a 100 μm coating. The system was assembled, packed in a custom-designed column, and evaluated for alcohol recovery from methanol/water and *n*-butanol/water mixtures. The ZIF-8@Cu laminates exhibited high adsorption capacities: 0.19 g_MeOH_/g_ZIF‑8_, 0.26 g_*n*‑BuOH_/g_ZIF‑8_, and excellent selectivity toward alcohols (α_MeOH/H_2_O_ = 8.5; α_*n*‑BuOH/H_2_O_ = 68). Vapor-phase experiments showed dispersive effects in the elution curve, attributed to the intrinsic properties of ZIF-8 (S-shaped equilibrium isotherm) and mass transfer limitation caused by channel nonuniformities and inlet flow maldistribution. For both separation mixtures, the laminate system was regenerated within 2 h via thermal swing adsorption (TSA), thereby exhibiting the combined benefits of microporosity, low-pressure drop, mechanical stability, and efficient heat transfer. The adsorptive properties were further highlighted in liquid-phase separation, where the laminates selectively captured *n*-butanol from 2.0 wt % aqueous solution and were successfully regenerated via TSA. This study provides proof of concept for the application of MOF-coated metal laminates in multiple adsorption–desorption cycles, thus highlighting their potential for process intensification.

## Graphical abstract

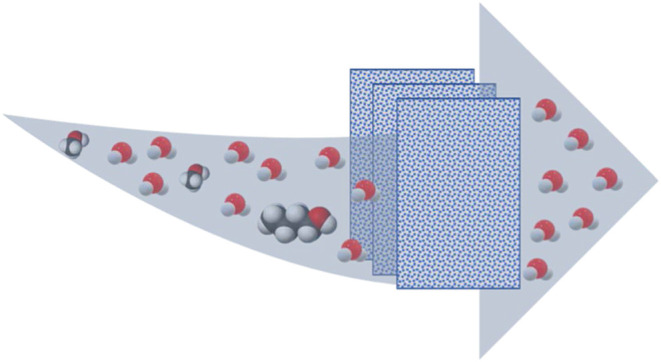

## 1 Introduction

Metal–organic frameworks (MOFs), also known as porous coordination polymers, are known for their highly ordered polymeric open-network structures formed via coordination bonds between metal-containing nodes and organic linkers.^1^ They exhibit high porosity, high specific surface area (1000–10,000 m^2^/g), tunable structure, modifiable functionality (postsynthesis), multivariate structures with multiple metals and/or organic linkers, and facile characterization by X-ray diffraction (XRD),^2^ which have prompted a myriad of potential applications such as gas storage,^3^ separation,^4^ catalysis,^5^ sensing,^6^ proton conduction,^7^ and biomedicine delivery.^8^ However, for their industrial applications, several important aspects need to be considered, such as long-term (hydrothermal) stability, ease of synthesis, cost, sustainable manufacturing, ability to regenerate, and so on.^2^ Another aspect that needs further attention is developing advanced solutions for shaping and structuring these materials into the optimal macroscopic form needed for their applications.

So far, different techniques like extrusion, spray drying, mechanical compaction, rolling granulation, embedded granulation, gel technology, and phase inversion have been used to process MOF powders into objects of millimetric dimensions such as extrudates, beads, and pellets.^9,10^ However, these shaping processes generally have a negative influence on the adsorptive properties. For instance, to achieve effective utilization, the MOF granules must possess sufficient bulk density, mechanical, chemical, and attrition resistance, and comparable performance w.r.t. the pristine MOF. Additionally, the high-pressure drop associated with the flow through a packed bed of beads or pellets, along with mass transfer limitation caused by slow diffusion in and out of the granules, can limit their performance, particularly in large-scale applications. Thus, shaping MOFs in more complex geometrical configurations, such as monoliths, honeycombs, laminates, foams, etc., is of considerable interest, which can demonstrate rapid process dynamics and fine-tuning mass and heat transfer properties.^10,11^

To date, several structured MOFs, such as thin films, foams, gels, paper sheets, monoliths, and hollow superstructures, have been synthesized.^9,10^ For example, thin MOF films on substrates can be obtained by employing methods like liquid-phase epitaxy (LPE),^12^ crystal growth,^13^ seeding,^14^ electrochemical deposition,^15^ layer-by-layer growth,^16^ Langmuir–Blodgett deposition,^17^ and solvent-free hot-pressing.^18^ Meanwhile, for foams and gels, dip-coating and deposition techniques, followed by freeze-drying or solvent-induced hardening, have been investigated.^19^ The most studied structure that presents certain advantages over others due to its high robustness, ease of handling, and low flow resistance is the MOF-based monoliths, which have been developed via fabrication techniques such as extrusion and pressing,^20^ gelation,^21^ coating,^22^ and three-dimensional (3D) printing.^23^

A laminate system is a simpler form of a monolith where the channels are replaced by one-dimensional (1D) slits.^24^ According to Rezaei and Webley, laminate systems are promising and can exhibit significantly better performance than a packed bed, but only if the pore width or the spacing between the laminates is below 0.2 mm.^25^ However, fabricating these systems has practical difficulties, such as maintaining small spacing and a challenging manufacturing process. In addition, most of the literature is available as patents and requires hands-on experience.^26–31^ The techniques described almost entirely rely on ex situ approaches, involving the coating of the adsorbent (zeolite) onto commercially available structures such as rotary cyclic displacement chambers,^26,31^ carbon cloths,^27^ metallic sheets,^28,32^ metal wires,^30^ and open-celled structures.^33^ An alternative to the coating technique, i.e., freeze-casting of aqueous suspensions of zeolite 13X powder, along with bentonite and polyethylene glycol, has also been described.^34^ Here, the authors prescribed a thermal treatment of the cast at 1053 K, which resulted in a mechanically stable 13X zeolite monolith with a laminated structure. Another approach that has been explored recently is obtaining laminates via extrusion and pressing of 13X zeolite paste (84% zeolite 13X content) and packing them into a micromilled aluminum mold.^35^ The authors evaluated three configurations through breakthrough experiments and compared them with a packed bed. The highlight of their work was the laminates with 1.2 mm thickness and 0.4 mm spacing that showed sharp breakthrough profiles and a 19% increment in the volumetric capacity with respect to the packed bed of pellets. However, a major drawback of the studies presented above was the use of bentonite clay as an inert binder. Meanwhile, the presence of such additives is necessary for mechanical stability. They can also dilute the active components, thus reducing the overall volume efficiency, and require high temperatures post-treatment, which is destructive for MOFs.^36^ In addition, they require uniform spacers. Born et al. designed a four-piece aluminum column with spacers milled in the side walls and assembled them into a column.^35^ In addition to rigorous design, the proposed layout offers limited material customization freedom. In other words, the column design is constrained by the laminate size, as extending the length or width of the laminates could affect their mechanical stability, reducing their ability to support their weight and maintain stable packing. Another drawback is the thermal insulation behavior of the extruded laminates. Adsorbents like zeolites and metal–organic frameworks (MOFs) tend to present intrinsic low thermal conductivity.^37–39^ For instance, pure zeolite 13X has a thermal conductivity of 0.08–0.13 W/(m·K),^40^ while for ZIF-8, it measures 0.326 W/(m·K),^37^ comparable to insulating materials like wood.^41^ In 3D-printed or extruded forms, this ranges between 0.1 and 0.9 W/(m·K).^39,42^ Poor thermal conductivity in separation applications can lead to localized overheating, compromising the structural integrity of the material, possible degradation, and reduced performance.^42^ Overcoming these drawbacks is essential to improve the performance and adaptability of laminate systems.

To address these limitations, an alternative approach inspired by the “LEGO concept” is proposed. A laminate system comprised of embossed/dented copper sheets, used as a support/substrate, can be stacked together. Following a direct in situ coating route,^43^ these embossed sheets were coated with ZIF-8 crystals and, later, assembled in an aluminum column designed in-house for laminate packing. Given the promising adsorptive behavior of ZIF-8 toward alcohol recovery from aqueous solutions,^44–46^ the developed ZIF-8 laminate system was subsequently evaluated for the recovery of methanol and *n*-butanol from aqueous solutions and humid vapor mixtures. This investigation aims to understand design trade-offs and quantify laminate system performance.

## 2 Experimental Section

In this section, the design and development of a laminate-based structured adsorbent are described. For information on materials and methods, please consult the Supporting Information (SI).

### 2.1 Fabrication of Laminate-Based Structured Adsorbents

The use of spacers in laminate systems is not mandatory, as metal laminates can also be shaped/structured into a specific form, like a corrugated sheet,^47^ and spiral wound.^32^ The structured laminate thus acts as a spacer, uniform throughout the system, providing an aperture for fluid to flow between the laminates and facilitating pressure equalization through the structure. Following the “LEGO” concept, in this work, embossed/dented copper laminates were fabricated (Figure 1d,1e).

**Figure 1:**
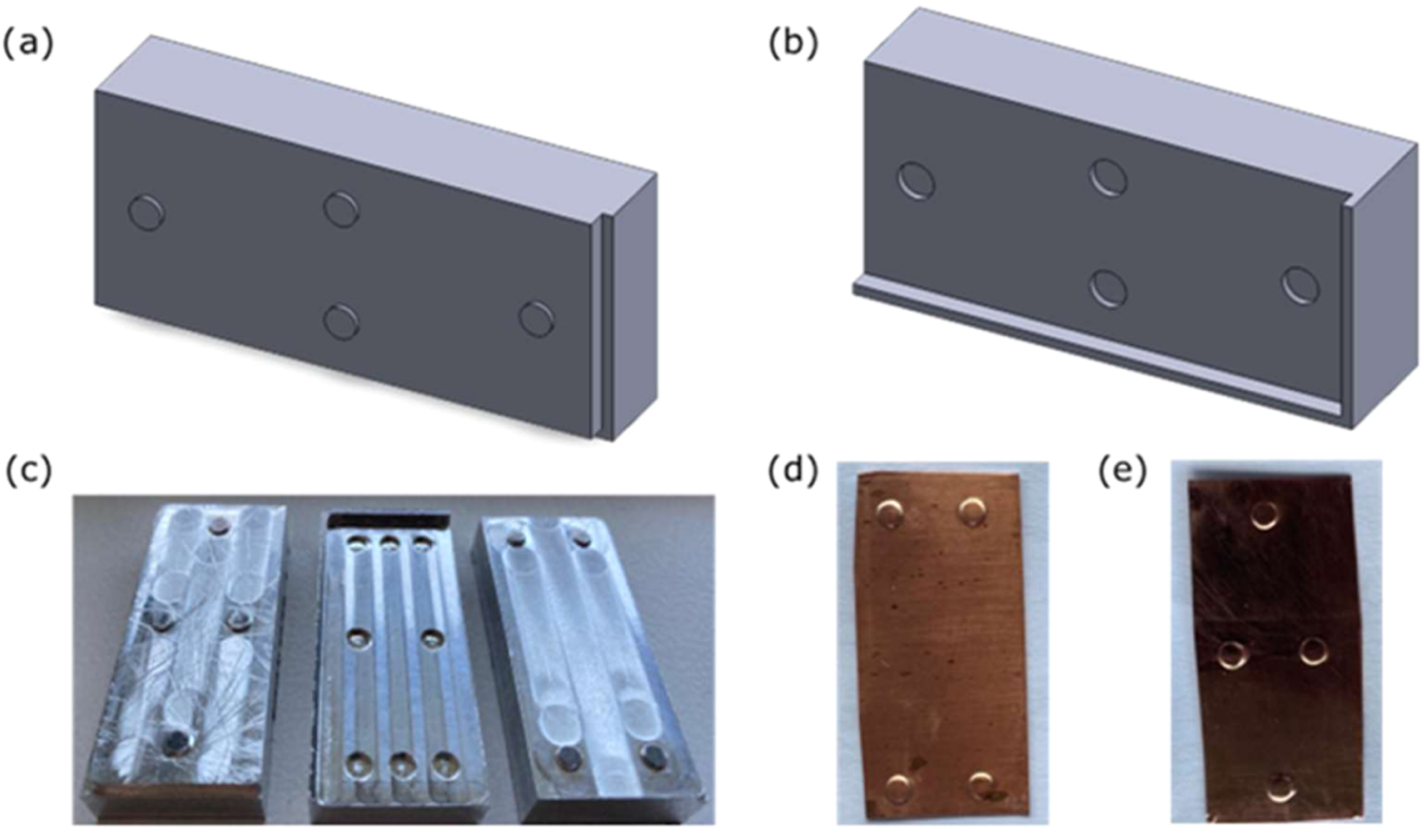
(a) Schematic of aluminum-based LEGO piece showing top cover (trimetric view) to obtain center-dented laminates, (b) schematic of aluminum-based LEGO piece (bottom base), (trimetric view), (c) photograph of in-house milled aluminum-based LEGO pieces used to fabricate dented copper pieces, (d) photograph showing embossed copper laminate at corner, and (e) photograph showing embossed copper laminate at center.

First, three aluminum-based LEGO pieces, including those with four cylinders embossed at corners and centers and four cylinders cut, were designed and developed, as shown in Figure 1c. The schematic of LEGO piece design is detailed in Figure 1a,b. Following the design, by precisely milling a meandering path, a cut was made using a CNC milling machine, and the aluminum LEGO pieces were fabricated (Figure 1c). Further information on the dimensions of these pieces is provided in the Supporting Information (SI). The role of the spacer between the laminates was played by a dent/embossing of 0.20 mm, which was produced by placing a copper laminate of size 4.70 × 2.10 cm^2^ between the LEGO aluminum pieces and pressing them under a hydraulic French Press (Piqua) at a pressure of 30 MPa. Two different designs were made, i.e., (a) corner-dented laminate (Figures 1d and S2a,b) center-dented laminate (Figures 1e and S2b). For stacking, these laminates were placed on top of each other to form a laminate system-based structured adsorbent.

### 2.2 Column Design

A column with dimensions 4.75 × 2.20 × 2.00 cm^3^ was hollowed out inside a larger aluminum piece measuring 7.50 × 4.50 × 2.40 cm^3^ for carrying out breakthrough experiments. Figure 2a depicts the schematic of the column, while Figure 2b shows a photograph of the in-house milled aluminum-based column. Figure 2c shows the schematic of the packing system. It includes fabricating the dented copper laminates with the LEGO aluminum pieces, coating them with ZIF-8 crystals, and stacking and inserting them into the aluminum column. Figure 2d illustrates the stacking of ZIF-8-coated copper laminates. Since the stacked laminates measured around 1.60 cm in height, two 2 mm-thick aluminum pieces were placed above and below to secure the packing.

**Figure 2:**
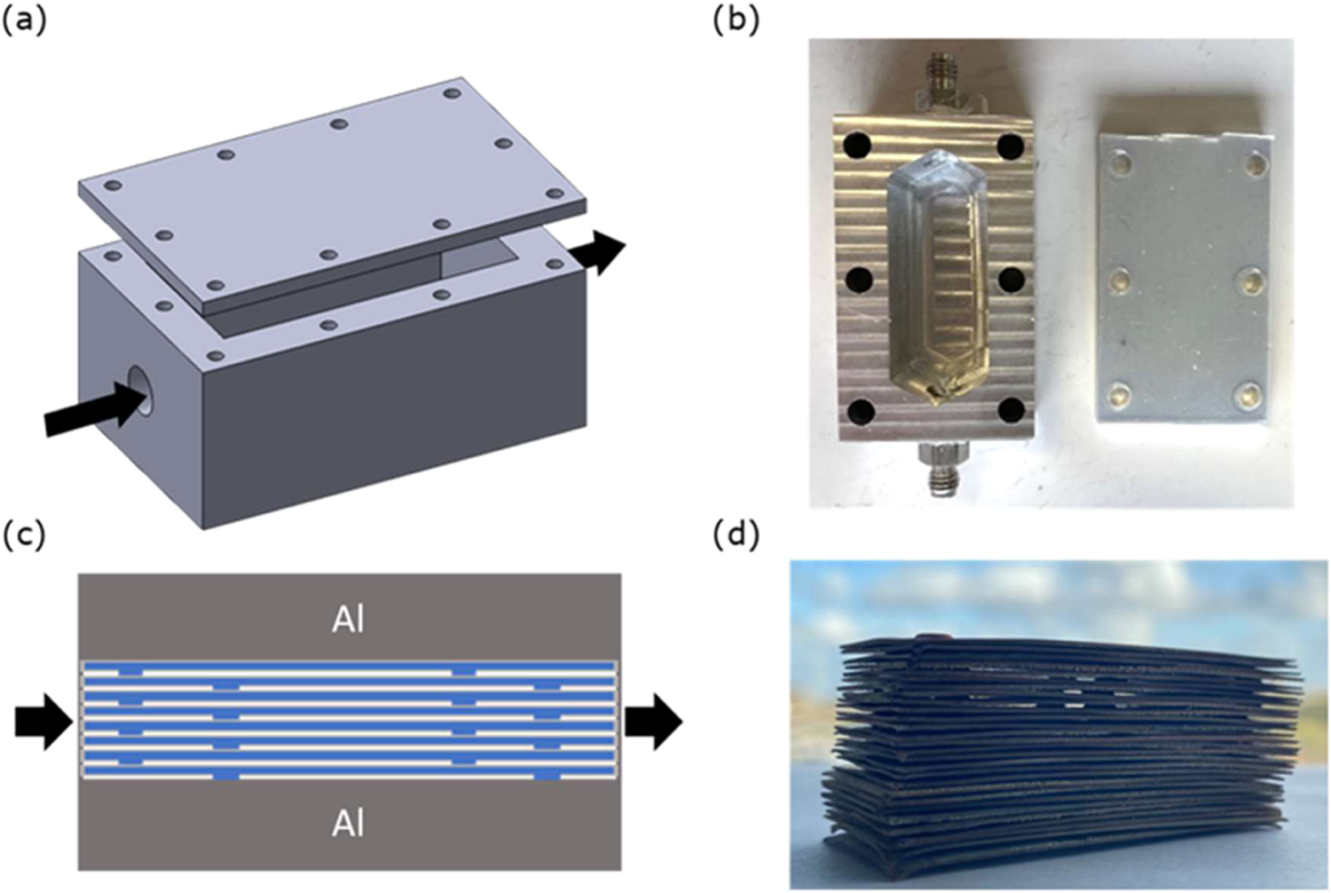
(a) Schematic of the column prepared for the laminate-based structured adsorbent (black arrow shows the gas inlet), (b) photograph of in-house milled aluminum-based column for breakthrough experiments, (c) schematic showing the packing of the embossed ZIF-8-coated copper laminates, and (d) photograph showing the stacking of the ZIF-8-coated embossed copper laminates in the open air.

## 3 Results and Discussion

In this section, the synthesis and characterization of the ZIF-8-coated laminate is described, followed by the performance evaluation of this developed laminate system for alcohol recovery in both vapor and liquid phases.

### 3.1 ZIF-8 Coating on Copper Laminates: Synthesis and Characterization

The ZIF-8 crystal coating on the embossed copper laminates (ZIF-8@Cu) was carried out following the synthesis method described in our previous work.^43^ In brief, rapid synthesis of ZIF-8 crystals can be achieved at room temperature by incorporating copper ions as a triggering agent/comodulator, along with sodium acetate as a modulator. Based on this knowledge, the growth of ZIF-8 crystals on both sides of copper laminate (ZIF-8@Cu) was achieved at room temperature following a direct in situ route. The process involved immersing a copper laminate in a reacting mixture composed of molar ratios of Zn­(NO_3_)_2_·6H_2_O/CH_3_COONa/C_4_H_6_N_2_/CH_3_OH = 1:4:2:200 at room temperature. After 24 h, the laminate was removed, washed with methanol and acetone to remove residual reagents and/or loosely adhered coatings, and dried at 363 K for 16 h. Figure 3 shows photographs of the copper laminate (embossed at corners) before and after the ZIF-8 coating, along with scanning electron microscopy (SEM) images of the ZIF-8-coated copper laminate and an X-ray diffraction (XRD) pattern of the ZIF-8 scratched from the copper laminate. A clear coating of a white powder can be seen on the copper laminate after the coating (Figure 3b). A SEM image of the top view reveals a uniform and continuous coating formed of cubic crystals with a size of around 8–10 μm (Figure 3c). The cross-sectional view shows a coating thickness of about 100 μm (Figure 3d). X-ray diffraction (XRD) analysis of the scratched coating confirms the material as ZIF-8 (Figure 3e) with all major peaks (7.4, 10.4, 12.7, 14.7, 16.4, 18.0°) matching with those reported for ZIF-8 ^43^ were observed. For more details on synthesis and characterization, consult our previous works.^43,48^

**Figure 3:**
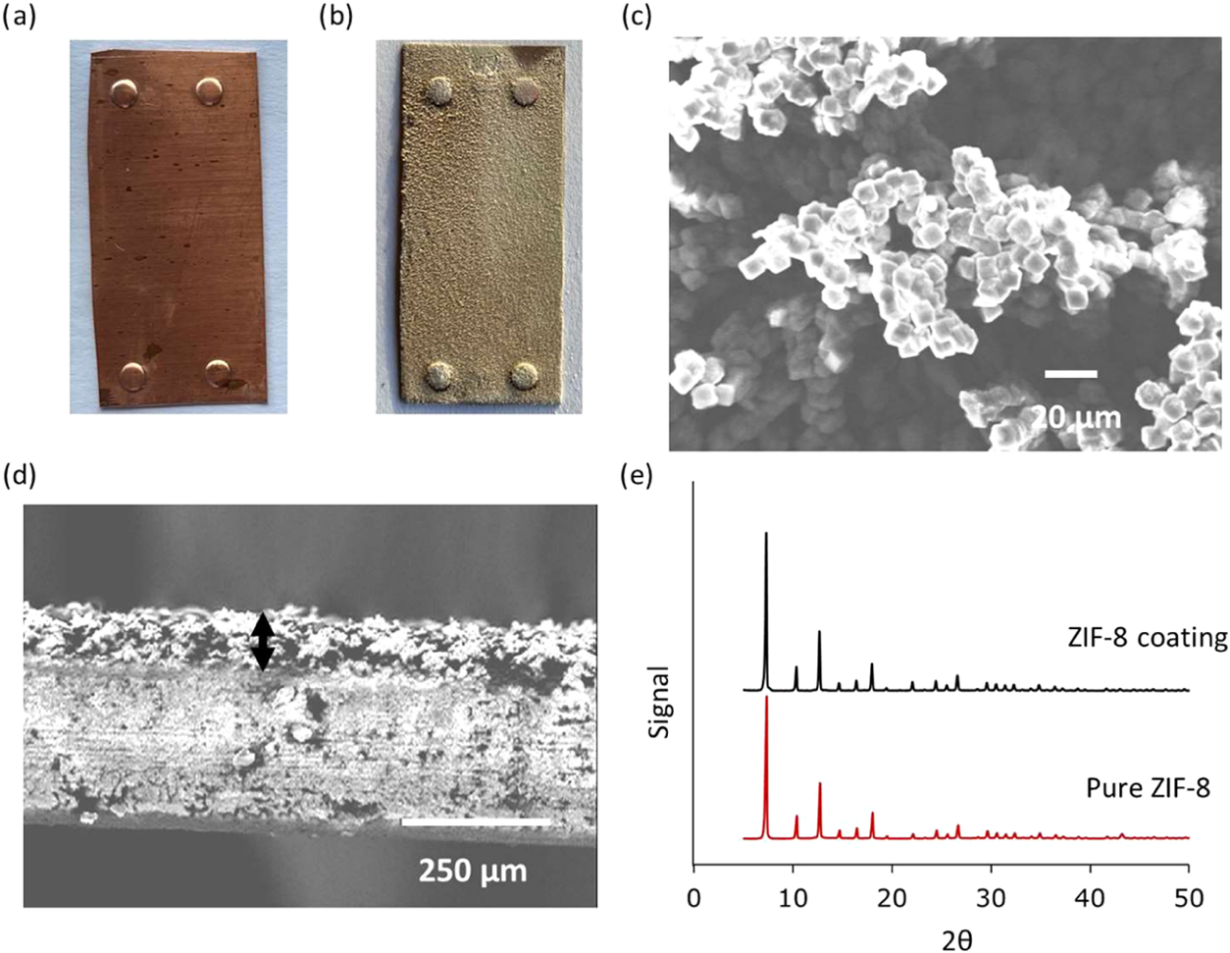
(a) Photograph of uncoated copper laminate embossed at corners, (b) photograph of ZIF-8-coated copper laminate embossed at corners, along with its (c) SEM image (top view), (d) SEM image (cross-section), the black arrow shows the ZIF-8 coating on copper surface (thickness ∼100 μm), and (e) XRD pattern of the coating compared with XRD pattern of pure ZIF-8.

Figures S4 and S6 display the Ar and N_2_ adsorption isotherms of the ZIF-8-coated copper laminate and commercial ZIF-8 purchased from BASF as a reference. The ZIF-8-coated copper laminate exhibited a characteristic double-stepped sorption behavior and a hysteresis loop in the desorption branch for both Ar and N_2_ adsorption isotherms, closely resembling the reference material. The two steps seen in the adsorption isotherms can be linked to the flexibility of the ZIF-8 linkers,^49^ and the hysteresis loop is associated with structural expansion and contraction of the framework.^50^ A change in the position and shape of the hysteresis loop was observed, which could be related to the energetics of the linker rotation process.^51^ The overall sorption behavior confirmed the retention of the intrinsic properties of ZIF-8; however, the amount of gas adsorbed by the ZIF-8-coated copper laminate varied from that of the reference ZIF-8. This is because the amount of ZIF-8 crystals grown on the copper laminate sample could not be precisely quantified due to partial copper dissolution during the synthesis process.^43^ This was observed from the change in color of the synthesis mixture from transparent to blue, indicating copper leaching into the solution (Figure S3b). Thus, to calculate the amount of ZIF-8 coating on the laminate surface, the micropore filling volume at *P*/*P*_o_ = 0.02 of both samples, i.e., ZIF-8 coated copper laminate and ZIF-8 crystals from BASF, was compared. The average amount of ZIF-8 coating per surface area (cm^2^) of the copper laminate was calculated to be 0.0027 g, with an estimated thickness of 100 μm (Tables S2 and S3). The calculated coating thickness corresponds to the SEM-measured thickness (Figure 3d).

Next, vapor-phase isotherms of *n*-butanol, methanol, and water were measured on the scratched ZIF-8 coating from copper laminate at 40 °C (Figure 4). As expected, the hydrophobic behavior of ZIF-8 can be observed in the isotherm of water. Meanwhile, an S-shaped isotherm, typical for the adsorption of short alcohols on hydrophobic adsorbents,^52^ was observed for guest alcohol molecules, i.e., methanol and *n*-butanol. The ZIF-8 coating adsorbed approximately 0.25 m_methanol_/g_ZIF‑8_ and 0.31 g_*n*‑butanol_/g_ZIF‑8_ at *P*_v_/*P*_sat_ = 0.45. This finding is in agreement with the literature.^44^

**Figure 4:**
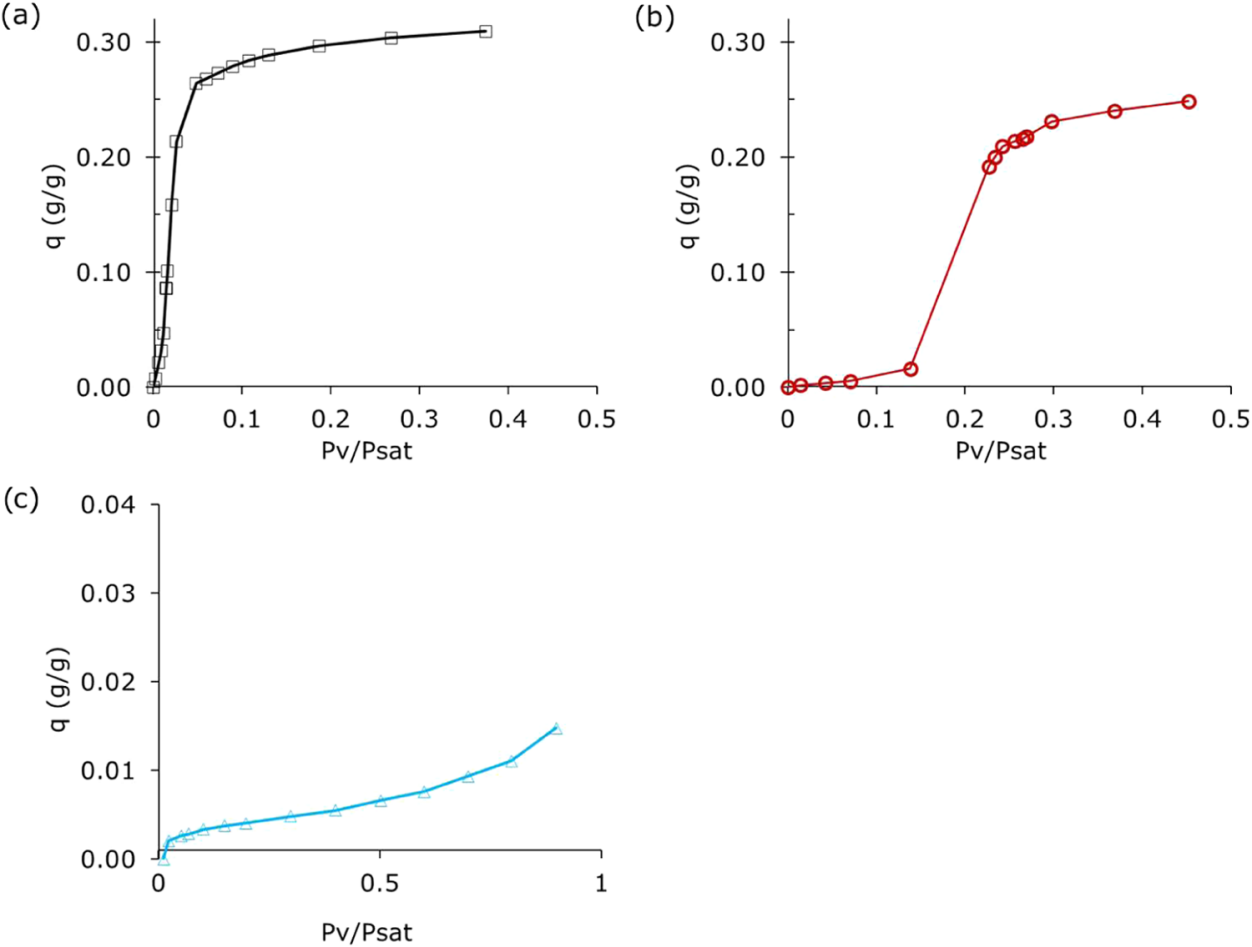
Isotherm of (a) *n*-butanol, (b) methanol, and (c) water vapor measured at 40 °C on ZIF-8 crystals scratched from the copper laminate.

Lastly, the mechanical and thermal stability of the ZIF-8-coated laminates and the laminate system was evaluated. First, five ZIF-8@Cu laminates were subjected to 1 h of sonication following thermal stress, in which the samples were rapidly heated from 25 to 250 °C. These tests were performed before and after the vapor-phase breakthrough experiments and again after the final liquid-phase adsorption test. In all cases, the laminate weights remained constant within the microbalance margin of error (±0.0001 g). During the packing and unpacking of the laminates for liquid-phase separation, a small amount of powder was observed inside the column. However, the overall mass loss remained within the balance’s margin of error, indicating negligible adsorbent loss. In an additional test performed after 120 days, the packed column was subjected to 1 h of sonication. The ZIF-8 coatings showed no visible damage or surface erosion (Figure S9b). A minor amount of detached powder was observed visually, corresponding to ∼1.2 wt % of the total ZIF-8 content. To confirm that the separation performance remained unaffected, vapor-phase *n*-butanol adsorption isotherms were measured at 40 °C using ZIF-8 coating scratched from the laminate after 120 days postsynthesis (Figure S9d). The isotherm profile remained consistent and in agreement with the literature.^44^ These results support the long-term stability of the ZIF-8-coated laminate system-based structured adsorbent for adsorptive separation via thermal swing adsorption (TSA). Here, the copper laminate not only provides mechanical support but also offers high thermal conductivity, acting as a thermal conductor and, thereby, addressing the heat insulation issues commonly observed in extrusion-based monoliths.^35,53^

### 3.2 Vapor-Phase Breakthrough Experiments

#### 3.2.1 Methanol/Water Mixture

Methanol is a versatile chemical feedstock that serves as an efficient energy-storage material and is easy to store and transport due to its liquid state. A promising approach for methanol synthesis is via CO_2_ thermocatalytic hydrogenation. However, water is produced as a byproduct in this reaction, which can deactivate catalysts due to sintering.^54^ Moreover, high-purity methanol is essential for its use in various applications such as fuel, chemical synthesis, and pharmaceuticals.^55^ To address this, the separation performance of the ZIF-8 coated laminate-based structured adsorbent was evaluated for methanol recovery from aqueous mixtures. Vapor-phase breakthrough experiments were conducted using a vapor mixture composed of 0.09 bar of methanol and 0.06 bar of water, with He as carrier gas at a flow rate of 12 NmL/min. This composition was chosen to simulate the mixture obtained after the thermocatalytic CO_2_ hydrogenation, as reported by Wu et al.^54^

The concentration profiles of MeOH/H_2_O vapor mixtures eluting from the laminate-based ZIF-8-coated structured adsorbent during adsorption and desorption are displayed in Figure 5 (time in hours), while Figure S8 provides a zoomed-in view of the initial stage of the adsorption cycle. During adsorption, water elutes immediately, with breakthrough occurring within the first 3 min and reaching its feed concentration in about 2 h (Figure S8). Methanol, on the other hand, shows a 2-step profile due to its isotherm sigmoidal shape (discussed below). The initial breakthrough is seen after about 10 min, with the saturation following after around 8 h. The amount of methanol adsorbed was ∼0.19 g_MeOH_/g_ZIF‑8_, with a selectivity (α_MeOH/H_2_O_) for MeOH over water of around 8.5. This capacity corresponds to approximately 76% of the equilibrium capacity (Figure 4b). In terms of volumetric capacity, the laminate system presented an adsorption capacity of 0.015 g_MeOH_/cm^3^.

**Figure 5:**
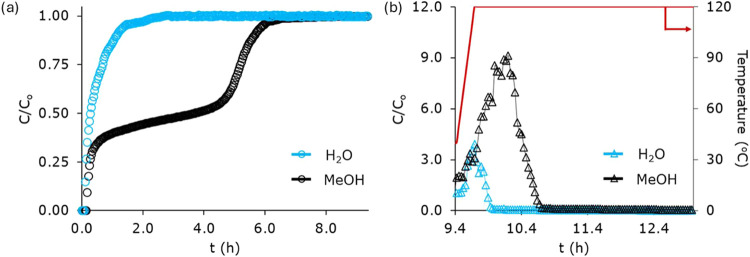
(a) Adsorption breakthrough profiles of methanol/water mixture on laminate-based ZIF-8-coated structured adsorbent (20 laminates) at 40 °C. The mixture is composed of 0.09 bar methanol and 0.06 bar water, with a total carrier gas (He) flow rate of 12 NmL/min. (b) Desorption profile of methanol/water mixture on laminate-based ZIF-8 coated structured adsorbent obtained by heating the column at 120 °C (5 °C/min) and purging the column with inert He gas at a flow rate of 12 NmL/min.

After the adsorption step, thermal regeneration of the ZIF-8-coated laminates was performed. The standard regeneration program consists of flushing the column with He gas at the same flow rate as during the adsorption step, i.e., 12 NmL/min, together with controlled heating of the column oven at 5 °C/min to 120 °C. The temperature was held constant for 5 h, followed by cooling to the experimental temperature. Figure 5b exhibits the desorption profile. Initially, both water and methanol are seen at the outlet when the temperature approaches 120 °C. The concentration of water rises until 18 min and then starts decreasing, with most of the water being desorbed within the first 25 min. Meanwhile, the concentration of methanol continues to rise, and desorption lasts approximately 80 min, indicating the potential to recover pure MeOH during the desorption step. In terms of methanol recovery, the system captures approximately 0.28 g (ca. 8.8 mmol) of MeOH per cycle, with each adsorption–desorption cycle lasting 101.6 min. This corresponds to a productivity of 3.12 kg_MeOH_/kg_ZIF‑8_/day.

Focusing on the shape of the breakthrough profile of methanol, two steps composed of broad “dispersive” and sharp “shock” profiles were observed. The shape of the breakthrough profile is dependent on the derivative of the adsorption isotherm. Thus, for Langmuir-type isotherm, i.e., an isotherm with decreasing derivative as a function of pressure, a sharp, shock-type profile will be obtained. Meanwhile, for an anti-Langmuir isotherm with an increasing derivative as a function of pressure, a broad, dispersive wave will be noted during the adsorption.^56^ Thereby, the double step seen in the breakthrough profile of methanol is due to the isotherm of methanol (sigmoidal or type V) (Figure 4b). This is in agreement with the findings of Cousin-Saint-Remi et al.^57^ and Claessens et al.,^58^ where the authors discuss the influence of the S-shaped isotherm of ethanol and *n*-butanol on the breakthrough curves for ZIF-8. According to the authors, at low partial pressure, only a dispersive wave is formed; meanwhile, at higher partial pressure, both shock and dispersive waves are observed.^58^ Similar reasoning on the effect of adsorption isotherm shape on breakthrough profiles is also provided by Helfferich and Carr.^59^

A key advantage of using a structured adsorbent over a traditional packed column is the possibility to operate at a higher flow rate while maintaining a low-pressure drop. To evaluate this factor, vapor-phase breakthrough experiments were performed at higher velocities, i.e., 3.9 cm/s (30 NmL/min) and 7.9 cm/s (60 NmL/min). The pressure drop across the column remained low, measuring 260 Pa/cm at 3.9 cm/s, and increased to 650 Pa/cm at 7.9 cm/s. In comparison, a packed bed of 250 μm pellets exhibited systematically higher pressure drop, i.e., 503 and 1004 Pa/cm, respectively, than the developed laminate-based structured adsorbent (Figure S7).

In addition to the low-pressure drop, higher flow rates can cause dispersion in the breakthrough profiles due to the channel nonuniformities and inlet flow maldistribution.^60^ Efficient separation requires a uniform inlet flow distribution. To assess this, the breakthrough profiles of methanol vapor at the column outlet were plotted in normalized time with respect to average breakthrough time (τ) (Figure 6). Irrespective of the flow rate, the double-step behavior, a consequence of the type V shape of the methanol isotherm on ZIF-8, was observed in all cases. However, the broadening of the profile was also seen with an increasing flow rate, possibly due to inhomogeneous flow distribution.^60^ In this study, the gas inlet was designed with an internal diameter of 1.55 mm; meanwhile, laminates have a rectangular cross-section of 2.10 × 1.20 cm^2^. In addition, the nonhomogeneous coating thickness (85–100 μm) and compressed laminate stacking could also contribute to channel nonuniformities. This, combined with flow maldistribution, may cause preferential flow through central channels and extra dispersion effects, leading to the broader breakthrough profiles observed. Similar findings have been reported in the literature. For instance, Claessens et al. investigated 3D-printed ZIF-8 monoliths for biobutanol recovery and concluded that the flow maldistribution caused by the partial blocking of the side channels for the 250 μm monolith led to a broadening of the *n*-butanol mass transfer zone at large carrier gas flow rates.^53^ In other studies, reported by Vortmeyer et al.^61^ and Roberts et al.,^62^ the broadening of concentration profile and breakthrough curves due to nonuniform flow distribution has been stated. Thus, both transport phenomena and the effect of the equilibrium isotherm could be in play. Overall, this case study presents a proof of concept for using a laminate-based ZIF-8-coated structured adsorbent to recover methanol from its mixture with water in the vapor phase.

**Figure 6:**
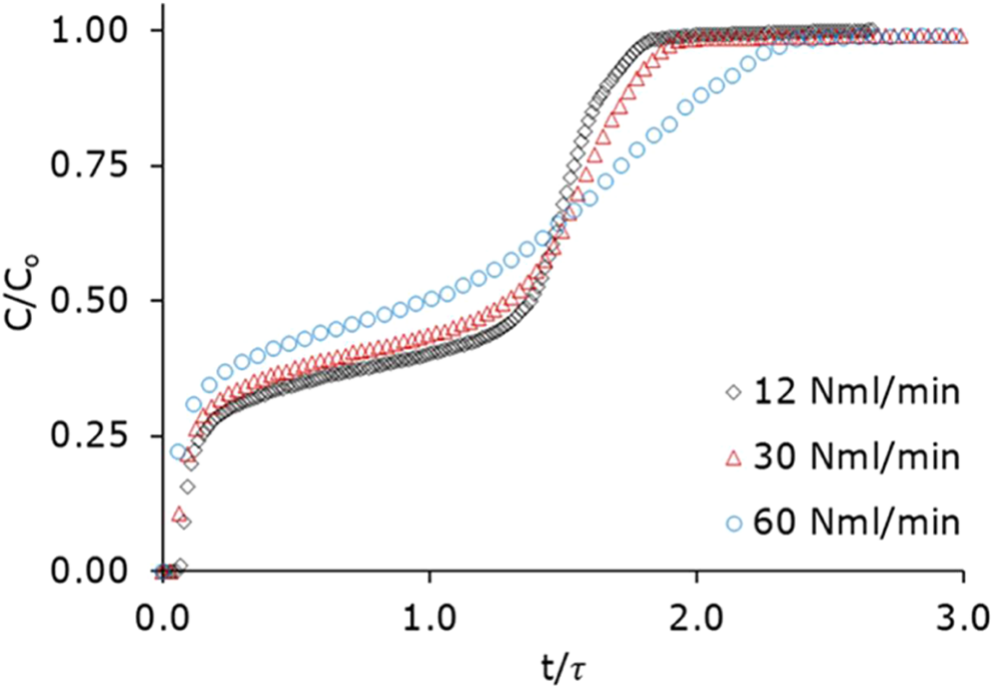
Adsorption breakthrough profiles of methanol vapor (0.12 bar) on laminate-based ZIF-8-coated structured adsorbent (20 laminates) with varying flow rate at 40 °C. The time scale is normalized to the average breakthrough time.

#### 3.2.2 *n*-Butanol/Water Mixture

Similar to methanol, another bioalcohol that has been pursued intensely is *n*-butanol. Due to its higher energy density and molecular similarity with gasoline, it is seen as a suitable replacement for conventional petroleum-based fuels. Properties such as lower vapor pressure and less corrosive behavior present it as a safe option for storage and transportation. Furthermore, low water solubility allows its use as fuel either via blending with diesel or without modifications.^63^ The production of biobutanol from renewable feedstocks through fermentation has gained attention as a sustainable alternative to petroleum-based fuels and chemicals. However, the downstream processing of biobutanol is energy-intensive (24 MJ/kg butanol)^64^ and costly.^65^ Alternative recovery methods, including liquid–liquid extraction, membrane extraction, pervaporation gas stripping, and adsorption, have been explored, with adsorption emerging as a particularly effective option, reducing energy consumption to 8 MJ/kg butanol.^64^ The recovery of biobutanol from the fermentation broth via adsorption can be achieved directly from the liquid fermentation broth or the gases produced during fermentation, with ZIF-8 identified as a leading adsorbent due to its high capacity and selectivity.^53^ Thus, the separation performance of the ZIF-8-coated laminate-based structured adsorbent for the recovery of *n*-butanol from the aqueous mixture was investigated via vapor-phase breakthrough experiments.

Figure 7a displays the concentration profiles of *n*-butanol/water (vapor) mixtures eluting from the laminate-based ZIF-8-coated structured adsorbent during adsorption (time in hours). As seen before for the methanol/water mixture, here also water is the first component detected at the column outlet, and *n*-butanol is the retained component, with an average breakthrough time of 8.01 h, an adsorption capacity of 0.26 g_*n*‑butanol_/g_ZIF‑8_ (∼93% of equilibrium capacity; *q* = 0.28 g_*n*‑butanol_/g_ZIF‑8_ at *P*_v,*n*‑butanol_ = 200 Pa), and a selectivity (α_*n*‑BuOH_) for *n*-butanol over water of around 68. Based on the bed density, i.e., the amount of ZIF-8 loaded per unit volume (∼0.08 g/cm^3^), the laminate system exhibited a volumetric *n*-butanol capacity of 0.020 g/cm^3^. This corresponds to about 81.3% of the capacity reported for a binderless ZIF-8 pellet-packed column by Claessens et al.^53^

**Figure 7:**
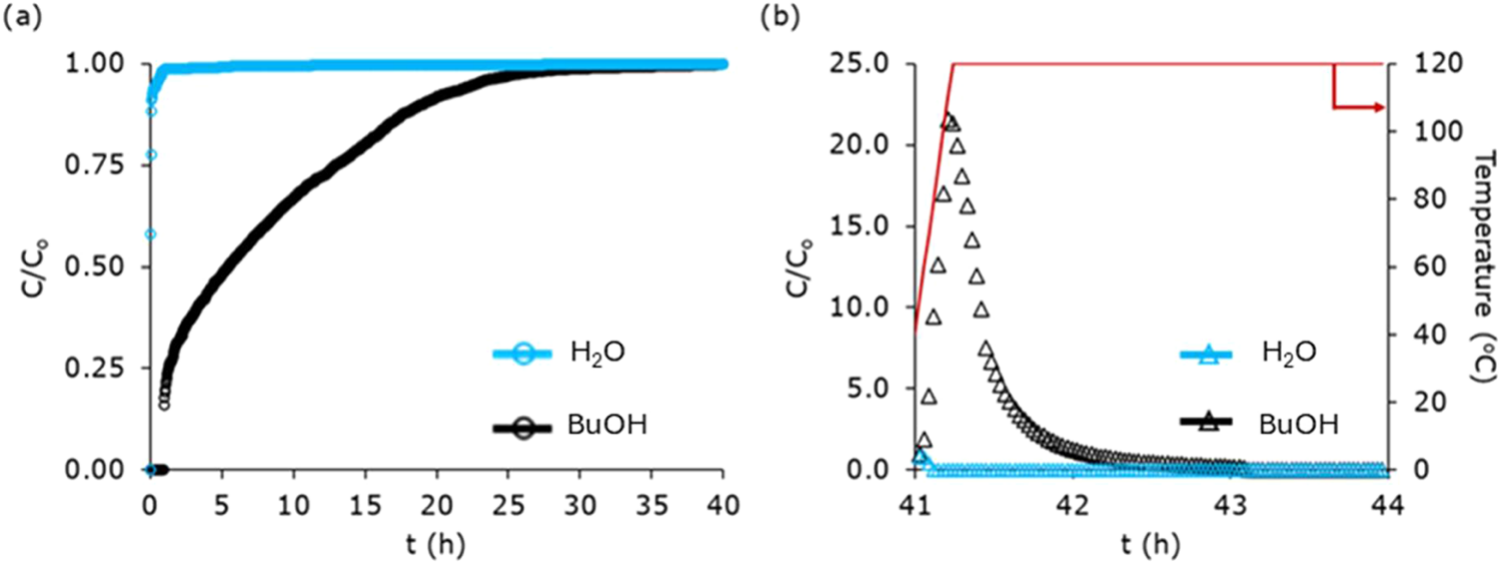
(a) Adsorption breakthrough profiles of *n*-butanol/water mixture on laminate-based ZIF-8-coated structured adsorbent (20 laminates) at 40 °C. The mixture is composed of 200 Pa *n*-butanol and 4075 Pa water, with a total carrier gas (He) flow rate of 123 NmL/min. (b) Desorption profile of *n*-butanol/water mixture on laminate-based ZIF-8-coated structured adsorbent performed by heating the column at 120 °C (5 °C/min) and purging the column with inert He gas at a flow rate of 123 NmL/min.

Focusing on the curve, a very dispersed breakthrough with a long tail was observed. This dispersive elution profile is understood to be the result of both experimental conditions and intrinsic properties of ZIF-8. As seen with the MeOH/H_2_O mixture at high gas velocities, the column design may cause maldistribution of flow at the inlet, which, combined with channel nonuniformities, lead to possible preferential flow through central channels and extra dispersion effects.^53^ In addition to these factors, low partial pressure of adsorbate (ca. 200 Pa *n*-butanol) and thick adsorbent coatings (ca. 100 μm) can also slow down the adsorption.^57,60^ Another factor that can affect the adsorption kinetics, particularly in the case of *n*-butanol adsorption by ZIF-8 crystals, is the size of ZIF-8 crystals (8–10 μm). As reported by Tanaka et al., the mass transfer rate of *n*-butanol in ZIF-8 is governed by both intercrystallite and surface resistance, with smaller ZIF-8 crystals (0.060–0.47 μm) exhibiting different adsorption kinetics compared to larger crystals (88 μm).^66^ The combination of these factors contributed to the dispersed nature of the breakthrough curve.

Following the adsorption step, regeneration was performed via thermal swing adsorption (TSA), using the same heating program as that described above for methanol. Figure 7b shows the desorption profile. As the temperature rises steadily toward 120 °C, a small amount of water at the outlet was observed initially. Meanwhile, for *n*-butanol, as the temperature rises to 120 °C, its concentration also increases, reaching a peak amount within the first 15 min and then decreasing. About 93% of *n*-butanol desorbed within the first 60 min, with complete desorption in 2 h. As a result, high-purity butanol could be recovered at a productivity of 0.15 kg_*n*‑butanol_/kg_ZIF‑8_/day, comparable to that calculated for 3D-printed ZIF-8 monoliths, 0.13 kg_*n*‑BuOH_/kg_ZIF‑8_/day.^53^

In addition to its competitive productivity, the ZIF-8@Cu laminate system offers a significant energetic advantage over other structured adsorbents, primarily due to its unique combination of a low-pressure drop and high thermal conductivity (Table 1). 3D-printed/extruded monoliths, such as those composed of 13X and ZIF-8, offer design flexibility and scalability. However, these monoliths are typically limited by poor thermal transport properties. For instance, thermal conductivity values for 13X-based structures range from 0.1 to 0.9 W/(m·K);^35,67–70^ meanwhile, 3D-printed ZIF-8 monolith show a thermal conductivity of around 0.33 W/(m·K).^53^ Additionally, pressure drops in these systems can be relatively high, with values such as 19,200 Pa/cm reported for 13X monolith with 800 cpsi.^70^ These characteristics can hinder effective heat transfer and increase the energy demand during regeneration, especially in thermal swing adsorption processes. Metal-based adsorbent systems like 13X-coated steel monolith^39^ and MIL-101-coated copper foam^74^ offer improved thermal conductivity, i.e., 50.2 and 0.86 W/(m·K), respectively, owing to their metallic supports. However, the ZIF-8@Cu laminate system significantly outperforms these materials, exhibiting a thermal conductivity nearly 5 times higher than that of the 13X-coated steel monolith.^39^ This enhancement is attributed to the difference in the thermal conductivities of bulk copper (400 W/(m·K)^76^) and steel (33.6 W/(m·K)^77^). The direct integration of the ZIF-8 layer with the copper laminate enables rapid and uniform heat distribution across the adsorbent bed, promoting efficient regeneration and potentially reducing energy consumption during cyclic operation.

**Table 1:** Properties and Comparisons of the Adsorbent Monolithic Structures

monolith types	thickness (μm)	pressure drop (Pa/cm)	thermal conductivity (W/(m·K))	refs
3D-print 13X	650	700	0.1	^67^
3D-print 13X	800	n/a	0.1	^68^
3D-print 13X	2200	n/a	0.9	^69^
13X monolith	n/a	19,200	0.1	^70^
13X laminates	1050	20	0.6^b^	^35^
mordenite	n/a	n/a	3.63	^71^
cordierite/13X	2.5	n/a	3.0	^72^
13X/aluminum foam	5000	n/a	2.89	^73^
ZIF-8 film	0.3	n/a	0.33	^37^
3D-print ZIF-8	250	n/a	0.33^b^	^53^
MIL-101/copper foam	800–1000	n/a	0.86	^74^
13X/steel monolith	11	4.6	50.2^a^	^39^
ZIF-8@Cu laminate	100	1004	250.2^b^	this work

a Thermal conductivity of steel monolith support.

b Thermal conductivity calculated by generalized Bruggeman formula^75^ (SI, Section 2.4).

### 3.3 Liquid-Phase Separation

In this part, ZIF-8-coated copper laminates were investigated for the recovery of *n*-butanol from an aqueous mixture containing 2.0 wt % *n*-butanol. In brief, five ZIF-8-coated laminates, each measuring 4.7 × 2.1 cm^2^, were placed in four vials containing 100 mL of the aqueous solution and shaken for a specific duration (see SI for a detailed methodology). Figure 8a illustrates the transient average *n*-butanol adsorbed amount (*q_t_*) recorded as a function of the contact time (*t*). Within 1 h, the amount of *n*-butanol adsorbed was around 0.23 g_*n*‑butanol_/g_ZIF‑8_, which reached a maximum of 0.27 g_*n*‑butanol_/g_ZIF‑8_ after 8 h. For regeneration, the copper laminates were dried by using filter paper and then placed in the column used previously for the vapor-phase experiments. The column was flushed with inert gas (He) at 100 NmL/min for 1 h, followed by thermal regeneration at 120 °C (10 °C/min) while continuing to purge with helium at the same flow rate. As shown in Figure 8b, no water peaks were detected; only *n*-butanol was observed at the outlet of the column, with 91% of *n*-butanol recovered in the first 3 h and full regeneration achieved after 250 min. To assess the adsorbent loss, the weight of the laminate sheets was measured. No significant loss was observed with the weight remaining constant within the margin of error of the microbalance (0.0001 g). In general, this approach of intermediate drying and flushing steps may seem tedious, but this approach significantly reduces the cycle time from ∼42 h seen in the vapor-phase experiment (Figure 7) to ∼6 h (Figure 8), considering a 1 h adsorption cycle. This allows the recovery of high-purity *n*-butanol with a productivity of 1.02 kg_*n*‑butanol_/kg_ZIF‑8_/day, representing a 7-fold increase compared to vapor-phase conditions. In practical applications, liquid removal could be achieved by flushing with air, further enhancing process efficiency.

**Figure 8:**
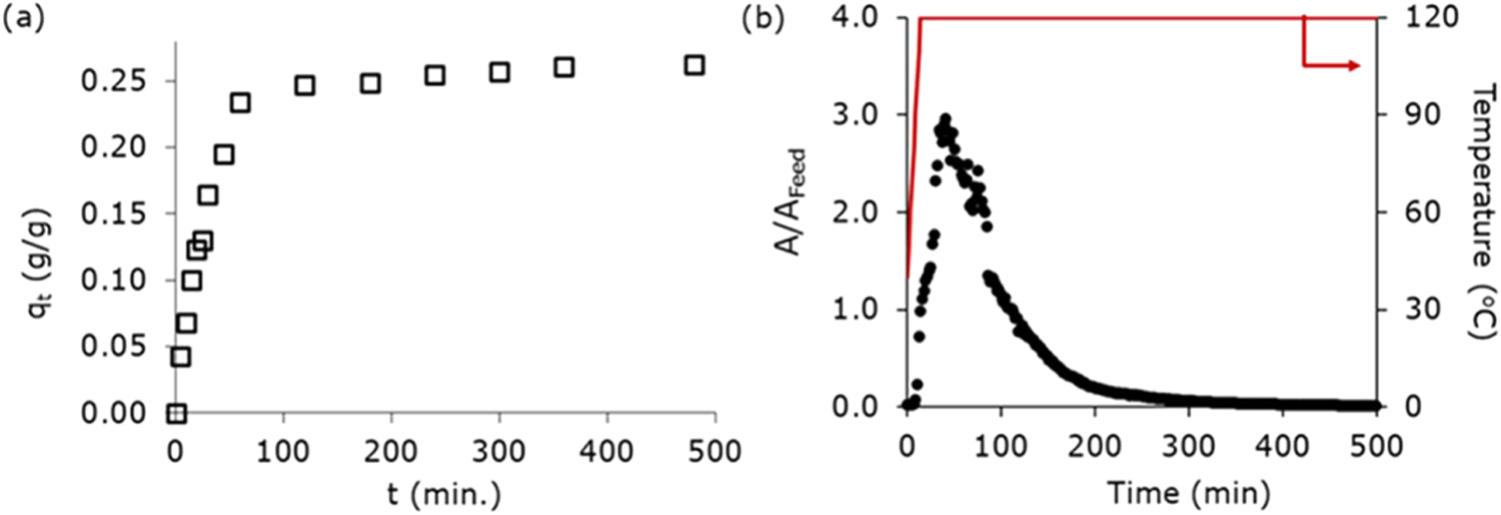
(a) Evolution of the butanol uptake (*q_t_*) on the ZIF-8 coated copper laminates with time. Batch experiment conditions: *V* = 100 mL, *T* = 298 K, average total ZIF-8 coating calculated = 0.53 g. (b) Regeneration profile of ZIF-8-coated copper laminates, obtained by heating the column at 120 °C (10 °C/min) and purging the column with inert He gas at a flow rate of 100 NmL/min. *A*/*A*_max_ corresponds to the ratio of the gas chromatography (GC) area of the peaks detected for *n*-butanol during regeneration and of the feed concentration during the vapor phase (*A*_max_).

## 4 Conclusions

In this work, an innovative approach toward the design and development of ZIF-8-coated metal laminate-based structured adsorbent for alcohol recovery in both vapor and liquid phases is presented. The laminate system was assembled using a modular LEGO approach, with the layout etched into aluminum pieces via CNC milling. The embossed/dented metal laminates were formed through a simple pressing technique and subsequently coated with ZIF-8 crystals by using a direct in situ method at room temperature. First, the stacked laminate-based structured adsorbent was evaluated for separating vapor-phase methanol/water and *n*-butanol/water mixtures. The findings confirmed that the adsorption properties of ZIF-8, i.e., high adsorption capacity and selectivity toward alcohols over water, were retained in the structured form. The dynamic breakthrough curves indicated dispersive effects due to the intrinsic property of ZIF-8 (S-shaped isotherm), low inlet partial pressure, thick ZIF-8 coating, and mass transfer effects caused by the channel nonuniformities and inlet flow maldistribution. For both mixtures, the laminate system was fully regenerated within 2 h via thermal swing adsorption (TSA), thus highlighting a coalesce of properties of both the MOF and copper laminate, i.e., microporosity, low-pressure drop, mechanical stability, and efficient heat transfer. The laminates also showed effective liquid-phase separation, selectively adsorbing *n*-butanol from a 2.0 wt % aqueous solution, followed by successful regeneration through TSA. Overall, this study highlights the successful design and development of metal laminate-based structured adsorbents that retained the adsorptive properties of ZIF-8 while offering process intensification through low-pressure drop and rapid regeneration.

## Supplementary Material



## Data Availability

The raw/processed data required to reproduce these findings cannot be shared at this time as the data also forms part of an ongoing study.

## Notes

Detailed description of materials and characterization methods, along with supporting results (PDF)
