# Supplementary material for: In Situ ZIF-8-Coated Copper Laminate System for Fluid-Phase Adsorptive Separation
Source: ACS Appl Mater Interfaces. 2025 May 15;17(21):30943–53. doi: 10.1021/acsami.5c04227 (PMC12123569; doi:10.1021/acsami.5c04227)
Supplement: Supplementary file 1 [file am5c04227_si_001.pdf]

SUPPORTING INFORMATION

In-Situ ZIF-8 coated Copper Laminate System for Fluid phase Adsorptive Separation

Ravi Sharma, Shiara Uyttersprot, Gino V. Baron and Joeri F.M. Denayer*

Chemical Engineering Department, Vrije Universiteit Brussel, B-1050 Belgium

*Corresponding author: Joeri.denayer@vub.be

1. Materials and Methods

1.1. Materials

Zinc nitrate hexahydrate ($Zn(NO_3)_2.6H_2O$, ≥98 %), sodium acetate (CH_3COONa, ≥98 %), 2-methylimidazolate ($C_4H_6N_2$, ≥98 %), methanol (CH_3OH, ≥99.8 %), n-Butanol ($CH_3(CH_2)_3OH$, ≥99.9 %) and copper foil (Cu, >99 %) were used. All the chemicals, except for copper foil, were bought from Sigma-Aldrich. Copper sheets were bought from Dejond. All chemicals were used without any further purification.

1.2. Characterization

Scanning electron microscope (SEM)

The samples were studied using a JSM-6000 plus JEOL device (under vacuum, circa. 10-6 Torr). None of the samples was pre-treated for SEM analysis.

Powder X-Ray Diffraction

X-ray diffraction data (XRD) were recorded with a Bruker D8 Advance Eco diffractometer using Cu Kα radiation (40 kV, 25 mA) with LynxEye XE-T (PSD opening 3.3°) detector in the range of 5–70° 2theta, with 0.015° step size and time 0.5s/step. In the current work, the XRD patterns were collected from the ZIF-8 coating scratched from the laminate. When the substrate is present, the characteristic Cu (111) reflection at ca 43.3° 2θ is clearly visible, as demonstrated in our previous study[1].

Thermogravimetric analysis

The thermal behaviour was explored via thermogravimetric analysis (TGA), using a SENSYS Evo instrument (SETARAM Instrumentation). About 5mg of material was placed in an aluminium crucible (S08/12797) and subjected to a gradual increase in temperature (1 K min-1) from 303 K to 673 K under a pure helium flow (100 N ml min-1). The weight loss was recorded in function of time.

Adsorption properties of ZIF-8 crystals: As-synthesized ZIF-8 crystals and Basolite Z1200 BASF (commercial ZIF-8)

Alcohols:

Adsorption isotherms of ZIF-8 for methanol and n-butanol were measured by the dynamic gravimetric method on a SGA-100H microbalance system (VTI Corporation, USA). A reservoir filled with alcohol is temperature controlled through Peltier elements. N_2 bubbling through the reservoir entrains the alcohol vapor. This vapor stream continuously flows on the sample positioned in a sample holder connected to the microbalance. During the measurements, the total pressure is

constant near atmospheric pressure and the adsorbate partial pressure is controlled by regulating the saturator temperature and/or the dilution rate. Low vapor pressures were obtained by diluting the saturator flow at the lowest saturator temperature with N_2 flow. About 10 mg of the sample was placed in a stainless-steel sample pan and positioned in the microbalance system. After activation by heating to 200 °C during 2h and at a heating rate of 2 °C/min under N_2 flow, adsorption isotherms of alcohols were determined at 313 K by weighing the adsorbate uptake at different partial pressures of the adsorbate. The equilibrium criterion was typically set at a mass change <0.0050 wt.% over a period of 10 min. The recorded temperature and dilution rate were converted to the adsorbate vapor pressure using the Antoine equation:

$$\log P = A - \frac{B}{C + T}$$

Where p is the vapor pressure, T is temperature, and A, B and C are species-specific constants

Table S1. Antoine equation constants[2].

	A	B	C	T_{min} ($\boldsymbol{K}$)	T_{max} ($\boldsymbol{K}$)
n-Butanol	4.64930	1395.140	182.739	310.18	411.26
methanol	5.20277	1580.080	239.500	262.59	356.00

P in mmHg and T in $°C$.[3]

The methanol, butanol, and water vapor isotherms were collected on the ZIF-8 coating scratched from the laminate due to the limitations of the instrument used. The isotherm measurements require a minimum sample mass of approximately 5 mg to ensure reliable data, which is difficult to achieve when using the ZIF-8@Cu laminate directly due to the relatively low mass of active material per unit area.

1.3. Vapor-phase breakthrough experiments

In order to investigate the separation of mixtures in dynamic conditions, breakthrough experiments were carried out. The column was designed specifically based on the dimensions of copper substrates i.e., stacking of dented copper sheets and copper laminates and fabricated in-house by precisely milling a meandering path on aluminium. The dimensions of the column were 4.75 x 2.10 x 2.00 cm^3. The coated Cu substrates were placed in between two aluminium pieces inside the column along with glass wool at the inlet and outlet and the column was closed by putting the lid on the column and screwing it tightly. Leaks were checked by putting the column inside a water bath and flowing compressed air through it. After the test, the column was re-opened, and the contents were found to be dry.

Breakthrough experiments of vapor phase mixtures were performed on an in-house built setup (Figure S1). The system consists of two bubbler evaporators, through which a carrier gas (He) was sent. The adsorbate vapour pressure at the column inlet can be controlled by controlling the evaporator temperatures and by diluting the generated vapors with extra carrier gas (He). Two mass flow controllers were used for the stream dilution with different range of flow rates i.e., 0 – 0.37 Nl/min and 0 – 0.8 Nl/min. During the experiment, the column was kept at constant temperature by external heating in a convection oven (HP 4890 GC oven). The mixture composition of the effluent stream was analysed by continuous injection of the carrier/vapor mixtures in a gas chromatograph (GC, HP6890, Agilent). The GC was equipped with Stabilwax capillary column of dimensions 0.25 mm i.d. and 30 m length (Restek) and an FID and TCD detector for the analysis of organic components and water, using He as carrier gas. In order to avoid condensation of vapor in the set-up tubing, all lines were heated to 70 ℃. For regeneration of the adsorbent, it could be performed in the setup by controlling heating of the column oven, while continuously flushing the column with He (inert carrier gas). As for the desorption, the concentration profiles can also be measured/monitored via GC analysis during regeneration. The ZIF-8 coated laminate system was studied for vapor phase breakthrough experiments using the mixtures: MeOH/H_2O and n-BuOH/H_2O and the ZIF-8 coated copper laminate was regenerated/desorbed on this set-up after liquid phase batch experiments.

To start the experiment, first the column was flushed with He at room temperature. After it was contacted with the desired mixture (composition) of adsorbate by changing the feed via multiposition valve before the column. Before each measurement, the ZIF-8 coated copper substrate packed in the column was regenerated at 150 ℃ for a least 60 minutes under He flow.

Breakthrough curves were generated by plotting the outlet concentration profiles of mixture components in function of time. From this data, the amount adsorbed, capacity (qi), of the different mixture components can be calculated via mass balance over the adsorption column, and the amount adsorbed of mixture components was calculated using the following equation:

$$q_i = \frac{\left(F_{feed}\tau_{ST,i} - \varepsilon V\right) .\ x_{feed,i}\,\rho_f}{m_{ads}} \qquad (1)$$

with F_{feed}, $\tau_{ST,i}$, ε, V, m_{ads}, $x_{feed,i}$, and ρ_f denoting the volumetric feed flow rate (ml/min), the average breakthrough time of component *i* (min), the bed porosity, total column volume (ml), the mass adsorbent (g), the mass fraction of the component *i* in the feed mixture (g/g), and fluid density (g/ml).

The average breakthrough time was calculated from equation 2:

$$\tau_{ST,i} = \int_0^\infty \left(1 - \frac{c_i}{c_{o,i}}\right) dt \quad (2)$$

with c_i and $c_{o,i}$ representing the column outlet concentration and the feed concentration.

The above equations are valid only under the assumptions that no change in fluid flow rate due to adsorption is observed. The adsorbate density is also assumed constant all over the column and same as feed mixture density. It also indicates that (for vapor systems) a negligible pressure drop over the adsorbent bed is assumed, considering the structured form.

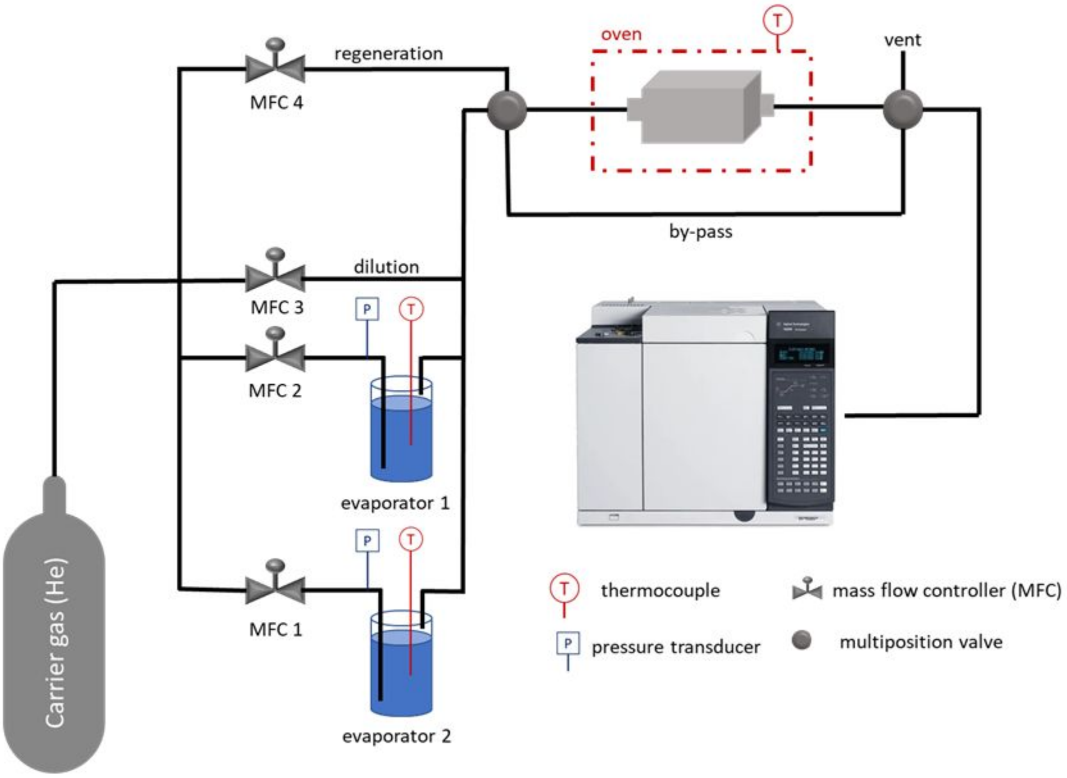

Figure S1. Schematic of the experimental setup to perform vapor phase breakthrough experiments. Helium (He) as carrier gas is sent through one or both evaporators to obtain a vapor stream. This vapor stream can further be diluted using another mass flow controller (MFC 3). The column is kept in a temperature-controlled oven and the effluent stream is analysed by GC. The adsorbent can be regenerated in-situ under continuous flow of carrier gas (He) by heating the column oven.

For vapor phase separation, considering the total mixture pressure at the column inlet, and the average of the total pressure for the two evaporators, the equation 1 was rewritten as:

$$q_i = \frac{\left(\dot{n}_{tot}^{mix} \cdot \tau_{ST,i} - \frac{\varepsilon V . P_{tot}^{mix}}{R . T_{ex}}\right) \cdot y_i^{mix}}{m_{ads}} \quad (3)$$

where $\dot{n}_{tot}^{mix}$ is molar flow rate of the mixture (mmol/min), , P_{tot}^{mix} is the total pressure of the mixture (Pa), R is the ideal gas law constant, q_i is the amount adsorbed (mmol/g), and T_{ex} is the experimental temperature (K) and,

$$\tau_{ST,i} = \int_0^{\infty}\left(1 - \frac{y_i^{outlet}}{y_i^{mix}}\right)dt \quad (4)$$

The selectivity (α_{ij}) of the adsorbent for an adsorbate *i* over adsorbate *j* can be calculated as [4]:

$$\alpha_{ij} = \frac{q_i/q_j}{y_i/y_j} \quad (5)$$

where q_i, q_j are amount adsorbed of *i* and *j* (mmol/g) and y_i, y_j are mole fraction of *i* and *j* in the mixture.

1.4. Liquid-phase batch experiments

Liquid phase batch experiments were performed to investigate the adsorption of n-butanol from aqueous mixtures. In this experiment, the adsorbed amount of an adsorbate is determined by bringing ZIF-8 coated copper laminate with a mixture of n-butanol/water (2 wt. %/98 wt.%). After a certain period of equilibrium time, the concentration of the mixture was measured again, thus allowing to determine the amount of n-butanol adsorbed. The concentration of 2 wt.% of n-butanol in the aqueous mixture was to mimic the fermentation broth where the amount of n-butanol after fermentation is around 1 – 2 wt.% [5,6].

The full experiment consisted of activating copper laminate coated with ZIF-8 by heating the sample to 150 ˚C at 1 ˚C /min. for 8 h. After activation, the samples were capped immediately in a vial and allowed to cool down to the room temperature. Afterwards, 100 mL of mixture solution was added to the vial containing the five ZIF-8 coated copper laminates. In total, 4 batch experiments were performed. The vials were later left to shake at shaker (KS 260, IKA) at 230 rpm. Using micropipette, 200 µL of sample was extracted after each hour and the composition of the liquid mixture was analysed via gas chromatography (GC) on an Agilent 7820A instrument equipped with a flame ionization detector (FID). A Solgelwax capillary column (0.25 mm i.d. – 30m length) supplied by Restek was used to separate the different mixture components.

The adsorbed amount was calculated by:

$$q_i = \frac{(C_i^o - C_i^e)\ .\ V_f^o}{m_{ads}} \quad (6)$$

where q_i, C_i^o, C_i^e, V_f^o, and m_{ads} are amount adsorbed of component *i* (g/g), initial concentration of component *i* (g/ml), equilibrium concentration of component *i* (g/ml), initial volume of liquid added to the vial (ml) and mass of the ZIF-8 coating on the copper laminate. To be mentioned here, the amount adsorbed calculated from the equation (16) corresponds to an excess adsorbed amount however, at low concentrations of n-butanol in a fermentation broth (~ 2 wt.%), the difference between the absolute and excess adsorbed amount is small[7].

2. Supplementary Results

2.1. Fabrication of 'Lego concept driven' laminate style structured adsorbent

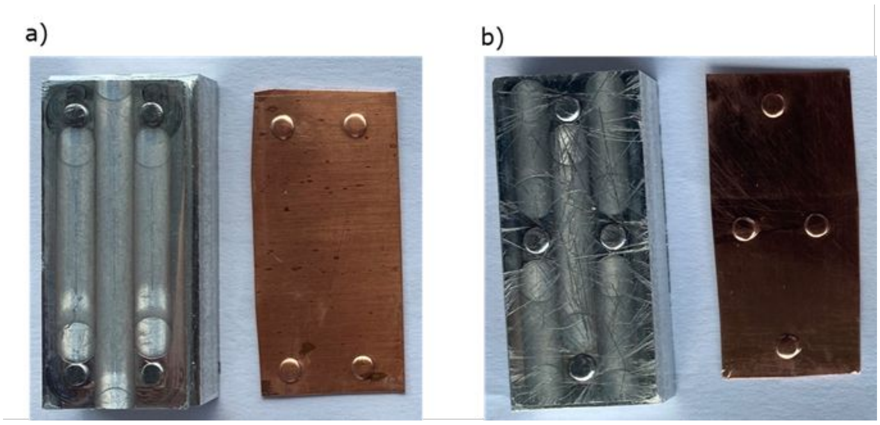

Figure S2. a) Photograph of in-house milled aluminium pieces with embossing on the corners (dimensions: 4.80 x 2.20 x 1.00 cm^3), photograph of embossed copper sheet at the corners after being pressed, and b) photograph of in-house milled aluminium pieces with embossing on the centres (dimensions: 4.80 x 2.20 x 1.00 cm^3), along with photograph of embossed copper sheet at the centre after being pressed.

2.2. Adsorption properties of ZIF-8 crystals: BASF (ref.) and collected from copper surface

For the argon and nitrogen sorption isotherms, as the amount of ZIF-8 coating on the copper foil was unknown, an assumption of 0.01 g of ZIF-8 coating was made in order to proceed with the analysis. With the correction in the weight, the thickness of the coating was calculated. For that, it was assumed that the amount of gas adsorbed by Cu foil itself is negligible and bulk density of ZIF-8 particle is 0.35 g/cc (ZIF-8 density as reported by BASF).

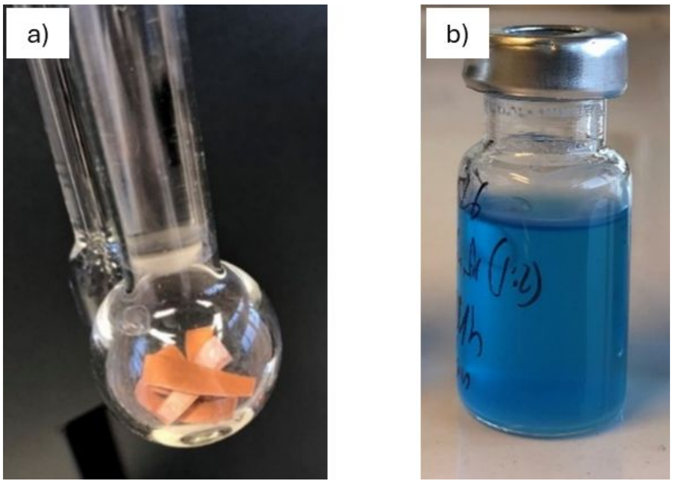

Figure S3. Photograph of a) ZIF-8 coated copper sheet as sample for adsorption studies and b) synthesis mixture after 24 h.

Argon

Argon adsorption isotherms of ZIF-8 crystals (reference from BASF) and ZIF-8 coated copper laminate sample at 87 K are exhibited in Figure S4. Both isotherms present double stepped sorption behaviour along with hysteresis in the desorption branch and is in overall agreement with data reported in the literature[8]. The difference in the amount of argon adsorbed is result of the mass assumption which was higher than the true amount of ZIF-8 crystals present on the copper sample with dimensions **3.05 x 1.11** cm^2 (Table S2).

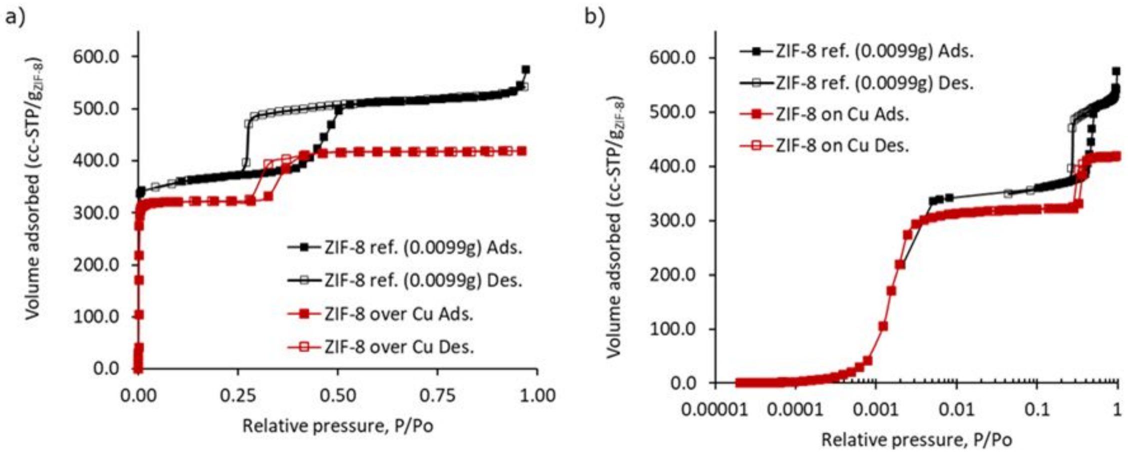

Figure S4. a) Argon (87 K) adsorption-desorption isotherms of ZIF-8 crystals (reference from BASF sample) and ZIF-8 coated copper laminate and b) Argon adsorption behaviour in logarithmic scale.

Table S2: Calculation of adsorbent mass and thickness on copper foil using the Ar (87 K) isotherm

	ZIF-8 ref.	ZIF-8 on Cu
Vol. adsorbed (cc-STP/g) at P/Po = 0.02	351.86	316.34
Ads. mass (g)	0.0099	**0.0089***
Thickness (μm)	--	**97.5***

*Calculated

$$\text{Ads. mass calculated (g)} = \frac{Vol.\,adsorbed\left(at\ \frac{P}{Po}=0.02\right)\,by\ ZIF{-}8\ coating}{Vol.\,adsorbed\left(at\ \frac{P}{Po}=0.02\right)\,by\ ZIF{-}8\ reference} \times ZIF-8\,ref.\,mass$$

$$Thickness\ calculated\ (\mu m) = \frac{Ads.\,mass\ (g)}{bulk\ density\left(\frac{g}{cm^3}\right)\times area\ (cm^2)} \times 10000\ {}^{\mu m}/_{cm}$$

With bulk density of ZIF-8 as 0.35 g/cm^3, the calculated amount of ZIF-8 coating on the sample was **0.0089 g**, and thickness was **97.5** $\boldsymbol{\mu m}$. The adsorbent (ZIF-8) superficial density was calculated as **0.0026 g/cm^2**.

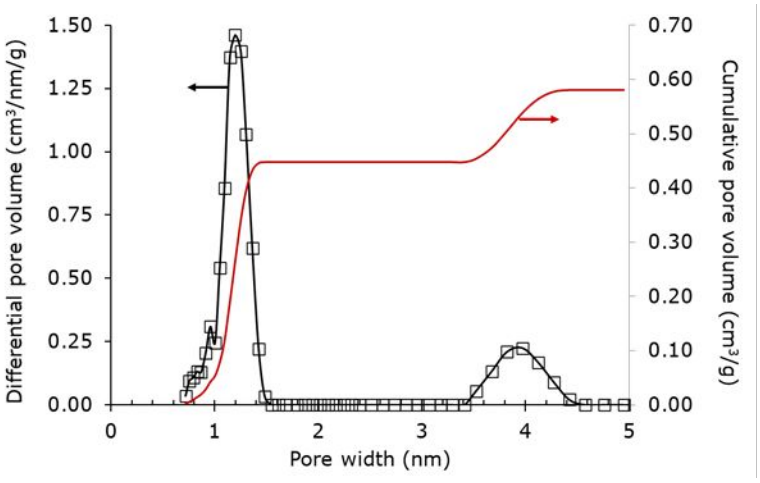

Figure S5. Micropore size distribution measured via Argon isotherm on a sample of ZIF-8 coated copper laminate.

The micropore distribution on ZIF-8 coated copper laminate exhibits peaks in between 0.793 – 1.484 nm and 3.411 – 4.575 nm.

Nitrogen

Nitrogen adsorption isotherms of ZIF-8 crystals (reference from BASF) and ZIF-8 coated copper laminate sample at 77 K are exhibited in Figure S6. Both isotherms present type-I adsorption isotherm, behaviour of ZIF-8 crystals revealing their microporous structure and is in overall agreement with data reported in the literature[8]. The augment in the amount of nitrogen adsorbed is result of the mass assumption which was lower than the true amount ZIF-8 crystals present on the copper sample with dimensions **3.74 x 1.22** cm^2 (Table S3).

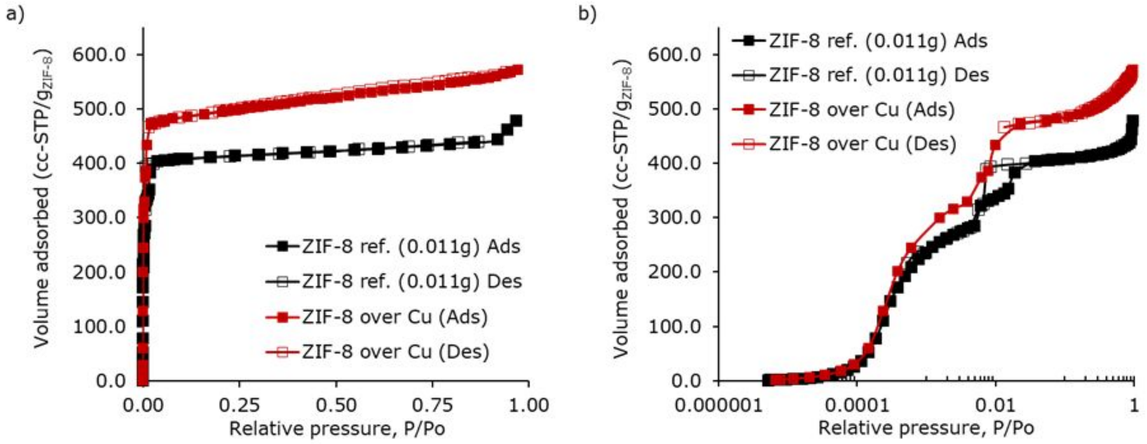

Figure S6. a) Nitrogen (77 K) adsorption-desorption isotherms of ZIF-8 crystals (reference from BASF sample) and ZIF-8 coated copper laminate and b) Nitrogen adsorption behaviour in logarithmic scale.

Table S3: Calculation of adsorbent mass and thickness on copper foil using the N_2 (77 K) isotherm

	ZIF-8 ref.	ZIF-8 on Cu
Vol. adsorbed (cc-STP/g) at P/Po = 0.02	402.91	472.80
Ads. mass (g)	0.011	**0.013***
Thickness (μm)	--	**102.3***

*Calculated

Ads. mass calculated (g) = $\frac{Vol.\,adsorbed\left(at\ \frac{P}{Po}=0.02\right)\,by\,ZIF{-}8\,coating}{Vol.\,adsorbed\left(at\ \frac{P}{Po}=0.02\right)\,by\,ZIF{-}8\,reference} \times ZIF - 8\,ref.\,mass$

$$Thickness\ calculated\ (\mu m) = \frac{Ads.\,mass\ (g)}{bulk\ density\left(\frac{g}{cm^3}\right)\times area\ (cm^2)} \times 10000\ ^{\mu m}/_{cm}$$

With bulk density of ZIF-8 as 0.35 g/cm^3, the calculated amount of ZIF-8 coating was **0.013 g**, and thickness was **102.3 μm**. The adsorbent (ZIF-8) superficial density was calculated as **0.0026 g/cm^2**.

2.3. Pressure drop

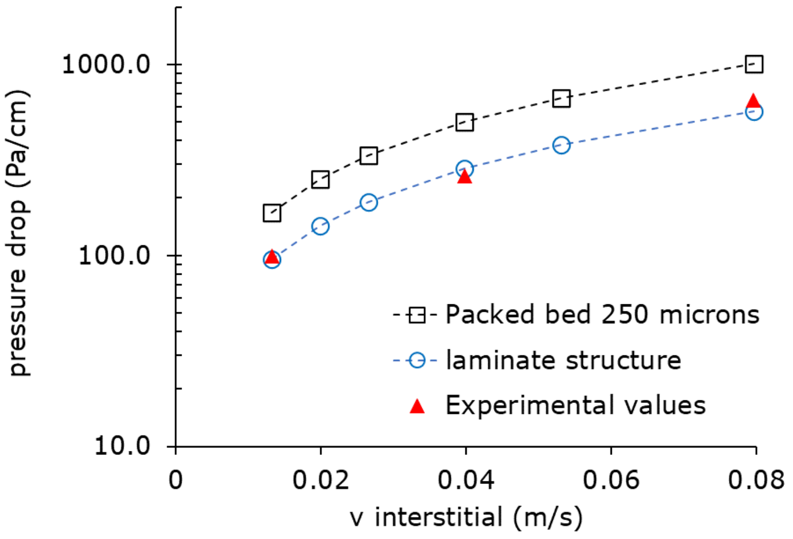

Figure S7. Comparison between the pressure drop over an adsorbent bed for pellets of average size 250 µm calculated from the Ergun equation, for laminate structure with 200 µm distance between the laminates calculated from the Hagen-Poiseuille equation, as described by Rezaei et al.[9], and the experimental pressure drop measured for the ZIF-8 based laminate system developed in this study. The experimental values coincide with the calculated pressure drop measured for the laminate structure.

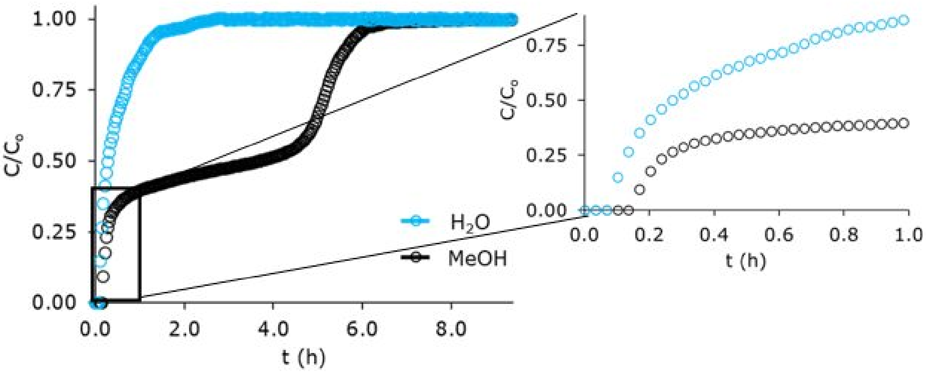

Figure S8. Adsorption breakthrough profiles of methanol/water mixture on laminate based ZIF-8 coated structured adsorbent (20 laminates) at 40 ℃. The mixture was composed of 0.09 bar methanol and 0.06 bar water, with a total carrier gas (He) flow rate of 12 Nml/min. The mixture composition was chosen to mimic a mixture obtained after the thermo-catalytic hydrogenation of CO_2, as reported by Wu et al.[10].

2.4. Thermal conductivity

To calculate the effective thermal conductivity (k_{eff}) of a composite material like a copper laminate coated with ZIF-8, the generalized Bruggeman formula was used. The formula is described below:

$$\sum_i \emptyset_i \frac{k_i - k_{eff}}{k_i + 2\, k_{eff}} = 0$$

where $\emptyset_i$ is the volume fraction and k_i is the thermal conductivity of each component[11]. The thermal conductivity of ZIF-8 was 0.326 W/m.K [12]and copper laminate was 400 W/m.K [13]. The thermal conductivity of 13X was 0.42 W/m.K[14] and of bentonite clay was 1.4 W/m.K[15].

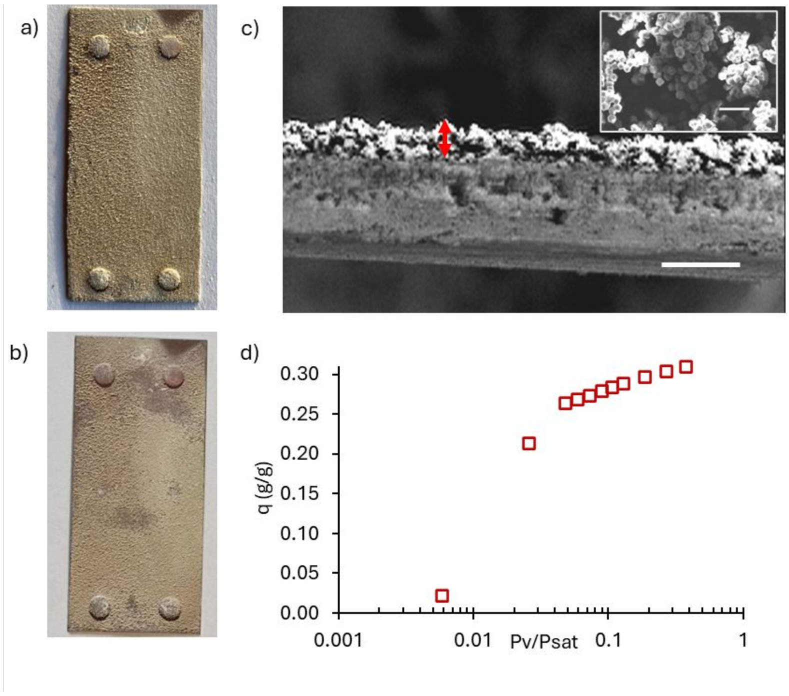

Figure S9. a) Photograph of ZIF-8@Cu laminate before the mechanical test (c.a. 2.6366 g), b) Photograph of ZIF-8@Cu laminate after the mechanical stability test conducted after 120 days (c.a. 2.6363 g), c) SEM image of ZIF-8@Cu laminate after the mechanical stability test (scale: 250 µm for cross-section and 20 µm for top view). The red line shows the ZIF-8 coating of thickness ~ 100 µm, and d) Vapor phase adsorption isotherm of n-butanol at 40 °C of ZIF-8 collected from ZIF-8@Cu laminate after the mechanical test. A capacity of around 0.31 $g_{n\text{-}butanol}/g_{ZIF\text{-}8}$ was measured at P_V/P_{Sat} = 0.37, which is in agreement with the literature[16].

References.

(1) Sharma, R.; Zhou, Z.; Themelis, T.; Van Assche, T. R. C.; Eeltink, S.; Denayer, J. F. M. Removal of Low Trace Ppb-Level Perfluorooctanesulfonic Acid (PFOS) with ZIF-8 Coatings Involving Adsorbent Degradation. *Langmuir* **2023**, *39* (9). https://doi.org/10.1021/acs.langmuir.2c03209.

(2) Poling, B. E.; Prausnitz, J. M. Properties of Gases and Liquids, Fifth Edition. *McGraw-Hill Education*. 2001.

(3) Shen, V. K.; Siderius, D. W.; Krekelberg, W. P.; Hatch, H. W. *NIST Standard Reference Simulation Website - National Institute of Standards and Technology*. NIST Standard Reference Database Number 173.

(4) Remy, T.; Peter, S. A.; Van Der Perre, S.; Valvekens, P.; De Vos, D. E.; Baron, G. V; Denayer, J. F. M. Selective Dynamic CO 2 Separations on Mg-MOF-74 at Low Pressures: A Detailed Comparison with 13X. **2013**. https://doi.org/10.1021/jp401923v.

(5) Amiri, H.; Karimi, K. Biobutanol Production. *Advanced Bioprocessing for Alternative Fuels, Biobased Chemicals, and Bioproducts: Technologies and Approaches for Scale-Up and Commercialization* **2019**, 109–133. https://doi.org/10.1016/B978-0-12-817941-3.00006-1.

(6) Abdehagh, N.; Tezel, F. H.; Thibault, J. Separation Techniques in Butanol Production: Challenges and Developments. *Biomass Bioenergy* **2014**, *60*, 222–246. https://doi.org/10.1016/J.BIOMBIOE.2013.10.003.

(7) Claessens, B. Enhancing Biobutanol Recovery via Adsorption: Adsorbents, 3D-Printed Monoliths and Unexpected Equilibrium Effects, VUB, Brussel, 2021, Vol. 7. https://doi.org/10.2/JQUERY.MIN.JS.

(8) Tanaka, S.; Fujita, K.; Miyake, Y.; Miyamoto, M.; Hasegawa, Y.; Makino, T.; Van Der Perre, S.; Cousin Saint Remi, J.; Van Assche, T.; Baron, G. V.; Denayer, J. F. M. Adsorption and Diffusion Phenomena in Crystal Size Engineered ZIF-8 MOF. *Journal of Physical Chemistry C* **2015**. https://doi.org/10.1021/acs.jpcc.5b09520.

(9) Rezaei, F.; Webley, P. Optimum Structured Adsorbents for Gas Separation Processes. *Chem Eng Sci* **2009**, *64* (24), 5182–5191. https://doi.org/10.1016/j.ces.2009.08.029.

(10) Wu, J.; Saito, M.; Takeuchi, M.; Watanabe, T. The Stability of Cu/ZnO-Based Catalysts in Methanol Synthesis from a CO2-Rich Feed and from a CO-Rich Feed. *Appl Catal A Gen* **2001**, *218* (1–2), 235–240. https://doi.org/10.1016/S0926-860X(01)00650-0.

(11) Ordóñez-Miranda, J.; Alvarado-Gil, J. J.; Medina-Ezquivel, R. Generalized Bruggeman Formula for the Effective Thermal Conductivity of Particulate Composites with an Interface Layer. In *International Journal of Thermophysics*; 2010; Vol. 31. https://doi.org/10.1007/s10765-010-0756-2.

(12) Cui, B.; Audu, C. O.; Liao, Y.; Nguyen, S. T.; Farha, O. K.; Hupp, J. T.; Grayson, M. Thermal Conductivity of ZIF-8 Thin-Film under Ambient Gas Pressure. *ACS Appl Mater Interfaces* **2017**, *9* (34). https://doi.org/10.1021/acsami.7b06662.

(13) J. R. Davis. *ASM Speciality Handbook, Copper and Copper Alloys*; 2001.

(14) Chao, J.; Xu, J.; Yan, T.; Wang, P.; Huo, X.; Wang, R.; Li, T. Enhanced Thermal Conductivity and Adsorption Rate of Zeolite 13X Adsorbent by Compression-Induced Molding Method for Sorption Thermal Battery. *Energy* **2022**, *240*. https://doi.org/10.1016/j.energy.2021.122797.

(15) Yoon, S.; Jeon, J. S.; Kim, G. Y.; Seong, J. H.; Baik, M. H. Specific Heat Capacity Model for Compacted Bentonite Buffer Materials. *Ann Nucl Energy* **2019**, *125*. https://doi.org/10.1016/j.anucene.2018.10.045.

(16) Cousinsaintremi, J.; Rémy, T.; Vanhunskerken, V.; Vandeperre, S.; Duerinck, T.; Maes, M.; Devos, D.; Gobechiya, E.; Kirschhock, C. E. A.; Baron, G. V.; Denayer, J. F. M. Biobutanol Separation with the Metal-Organic Framework ZIF-8. *ChemSusChem* **2011**, *4* (8), 1074–1077. https://doi.org/10.1002/cssc.201100261.
